# Sulfated Alginates as Heparin Analogues: A Review of Chemical and Functional Properties

**DOI:** 10.3390/molecules22050778

**Published:** 2017-05-11

**Authors:** Øystein Arlov, Gudmund Skjåk-Bræk

**Affiliations:** 1 Department of Biotechnology and Nanomedicine, SINTEF Materials and Chemistry, Richard Birkelands vei 3B, 7034 Trondheim, Norway; oystein.arlov@sintef.no; 2 Department of Biotechnology, Norwegian University of Science and Technology, Sem Sælands vei 6/8, 7034 Trondheim, Norway

**Keywords:** heparin, alginate, sulfated alginate, biomaterials

## Abstract

Heparin is widely recognized for its potent anticoagulating effects, but has an additional wide range of biological properties due to its high negative charge and heterogeneous molecular structure. This heterogeneity has been one of the factors in motivating the exploration of functional analogues with a more predictable modification pattern and monosaccharide sequence, that can aid in elucidating structure-function relationships and further be structurally customized to fine-tune physical and biological properties toward novel therapeutic applications and biomaterials. Alginates have been of great interest in biomedicine due to their inherent biocompatibility, gentle gelling conditions, and structural versatility from chemo-enzymatic engineering, but display limited interactions with cells and biomolecules that are characteristic of heparin and the other glycosaminoglycans (GAGs) of the extracellular environment. Here, we review the chemistry and physical and biological properties of sulfated alginates as structural and functional heparin analogues, and discuss how they may be utilized in applications where the use of heparin and other sulfated GAGs is challenging and limited.

## 1. Introduction

Glycosaminoglycans (GAGs) are a group of negatively charged linear polysaccharides found in virtually all animal tissues. The majority of GAG subtypes are associated with the plasma membrane via a protein core, referred to as proteoglycans, and are key components of the extracellular matrix (ECM) in providing structural support and hydration. The sulfated GAGs have additional vital roles in the development, maintenance, and pathophysiology of mammalian tissues, and may serve as receptors, co-receptors, and reservoirs through electrostatic interaction with proteins. Heparin differs from other GAGs in that it is primarily produced by mast cells, and is released from storage granules into the extracellular space by exocytosis. Heparin is a potent anticoagulant, and is widely used in the clinic as an intravenously administered blood thinner and as a coating material for medical devices. While structurally related to heparan sulfate (HS), heparin undergoes a greater degree of enzymatic modification during synthesis, resulting in highly diverse biological activities. Heparin has the highest sulfation degree of the GAGs, and a higher charge density than any known biopolymer, thus associating with a plethora of proteins including coagulation factors, growth factors, cytokines, adhesion proteins, and pathogen-related proteins.

The use of heparin for other biomedical applications than anticoagulation is however limited, partly due to rapid turnover in biological systems and risks of excessive bleeding upon administration. Heparin has a high degree of heterogeneity in its monosaccharide sequence and modification pattern, depending on the source, and has few options for structural customizability. This complicates tuning of the drug’s efficacy, as well as the characterization of biological activity and structure-function relationships required for exploring new potential applications. An additional aspect regarding widespread use of heparin is concerns regarding safety and sustainability in its production, as the most widely used derivatives of heparin are isolated from animal tissues [1]. For these reasons, over the last few decades numerous heparin derivatives and analogues from natural sources, chemical synthesis, and chemical and/or enzymatic functionalization of polysaccharides have been described [2,3,4,5]. Some of the heparin analogues described in the literature aim to emulate the anticoagulant properties of heparin, to provide more sustainable and safe drug manufacturing and/or allow greater control over pharmacokinetic and –dynamic properties. Others are directed toward novel pharmaceutical applications or as components in biomaterials, where the physicochemical properties and highly specific anticoagulating action of heparin pose limitations.

This review aims to present current knowledge and studies on sulfated alginates as novel heparin analogues, based on the authors’ own work and the available recent literature. We further wish to discuss future directions of this research, as well as areas of application where sulfated alginates can potentially provide a viable alternative to heparin or other sulfated GAGs and derivatives.

### 1.1. Heparin Molecular Structure and Physical Properties

**Figure 1:**
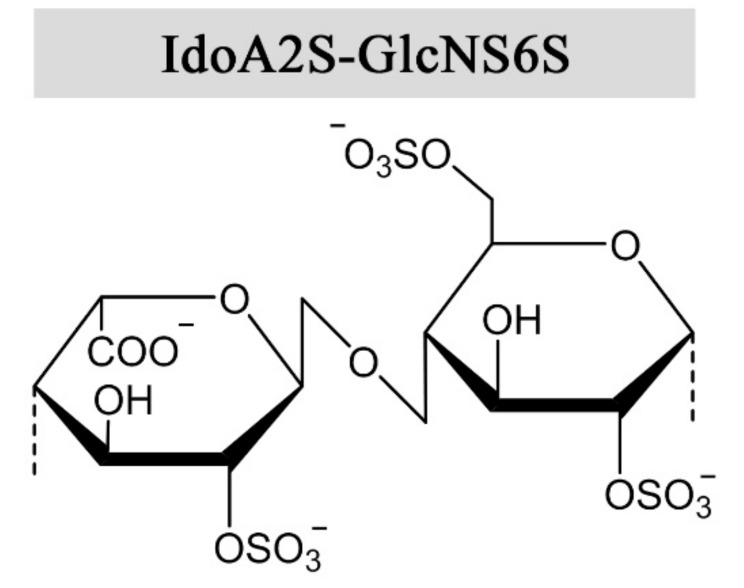
The prevalent disaccharide structure in heparin.

Heparin and heparan sulfate are synthesized as alternating copolymers of 1➜4-linked *N*-acetylglucosamine (GlcNAc) and glucuronic acid (GlcA). During synthesis, the nascent chain is modified by a series of enzymes in the Golgi apparatus, namely N-deacetylase-N-sulfotransferases, C-5 epimerases, and 2-O, 3-O, and 6-O sulfotransferases [6]. Epimerization of GlcA into iduronic acid (IdoA) confers structural flexibility to the polysaccharide chains, in that IdoA can assume one of three stable conformations (^4^C_1_, ^1^C_4_, or ^2^S_0_) depending on the modification pattern and induced effects upon protein interaction [7]. The expression of tissue-specific isozymes [8,9] and varying combinations of modifications confer a high degree of structural complexity and variability to heparin and HS, requiring a great effort for their functional and structural characterization. Heparin has a higher degree of sulfation and epimerization compared to HS, where the trisulfated disaccharide IdoA2S-GlcNS6S (Figure 1) constitutes 60–85% of the heparin chain, depending on the source [10,11]. However, there are no repeating sequences in heparin as the trisulfated disaccharides are interspersed by undersulfated residues of varying lengths. Heparan sulfate exhibits long unmodified regions between heparin-like motifs with high sulfation and epimerization degrees [12]. Epimerization of GlcA to IdoA has been demonstrated in vitro, using Glucuronyl C5-epimerase isolated from bovine tissue and the K5 capsular polysaccharide (GlcA-GlcNAc) [13]. To the authors’ knowledge, only one prokaryotic GlcA C5-epimerase has been identified and utilized for in vitro epimerization of K5. As the activity of the enzymes was relatively low, the results indicated that the native substrate differs from heparin and HS-like polysaccharides [14]. Of note, in vitro epimerization of GlcA is readily reversible, in contrast to the reaction in vivo due to subsequent O-sulfation following epimerization [13,15].

Commercial heparin is generally classified as unfractionated heparin (UFH, ~15 kDa), low molecular weight heparin (LMWH, ~6 kDa), or ultra-low molecular weight heparin (ULMWH, <2 kDa), where the most common ULMWHs are chemically synthesized oligosaccharides including the antithrombin (AT)-binding region [16]. Whereas chemical synthesis of heparin has had major limitations in terms of oligosaccharide length, recent advances have demonstrated production of up to 40-mers by iterative homologation of tetrasaccharides [17]. However, this approach generates repeating sequences, which are not found in native heparin.

### 1.2. Biological Properties of Heparin and Structure-Function Relationships

Heparin has potent anticoagulating properties and has widespread use in the clinic as an intravenously administered drug and as a coating material on medical devices. Its anticoagulating effect is primarily mediated by highly specific binding and activation of antithrombin, which in turn inactivates several proteases of the coagulation cascade, namely thrombin and Factors X, IX, XI, and XII [18].

Furthermore, heparin has demonstrated anti-inflammatory effects, presumably by multiple functions due to its broad substrate affinity. For instance, heparin has long been known to bind complement factors, where its anti-coagulating effects can supplement this effect through cross talk between the complement and coagulation pathways [19,20], as well as pro-inflammatory cytokines [21]. Binding of heparin to these proteins can exert an inhibitory effect through preventing receptor interaction, conformational changes, or proteolytic cleavage. An additional postulated mechanism is the binding of heparin to P- and L-selectins, inhibiting interaction with endothelial proteoglycans and thus adhesion and extravasation of leukocytes [22]. Heparin has also been evaluated as a potential antiviral drug due to association with viral capsule proteins [23]. Heparan sulfate and other GAGs on the cell surface can be utilized as a receptor for pathogens initiating adhesion and eventual cell entry, where soluble heparin can serve as an antagonist [24].

Heparin has been demonstrated to bind a plethora of proteins with varying interaction strengths. Asides from the well characterized AT interaction, the selectivity of heparin-protein interactions, and whether specific sequences are “programmed” into the heparin chains, remains a controversial subject. For a more comprehensive discussion of the topic, the reader is referred to the works of Lindahl and associates [25,26]. As a relevant point to the present review, it is evident that variance in monosaccharide sequences and modification patterns can have a large impact on protein interaction strength, tied to optimal alignment for ionic interactions and van der Waals forces [7]. For example, Hu and co-workers generated a HS disaccharide library, demonstrating that only 4 out of 48 disaccharides bind fibroblast growth factor-1 (FGF-1) with high affinity [27]. The presence of IdoA has been demonstrated to be critical for the interaction strength to specific proteins, where a large degree of chain flexibility can contribute to improved alignment between heparin and the protein surface, compared to a more rigid polysaccharide with equivalent charge. For example, the ^1^C_4_ conformation forms a kink in the heparin/HS chain which greatly enhances FGF interaction, but is not present in IdoA-containing dermatan sulfate due to the different interspersed monosaccharides and linkage patterns [28]. Studies have also shown the effect of various sulfation patterns in heparin on binding to P and L-selectins, revealing that 6-O-sulfation is critical for interaction with both selectins [29]. Furthermore, LMWH interacts less strongly with selectins compared with UFH, indicating an additional dependence of chain length, or that critical structural patterns are disrupted during fragmentation [30]. These examples emphasize that there are additional aspects beyond charge density that influence the biological activity of sulfated GAGs, which must be addressed in the design of functional analogues.

## 2. Sulfated Alginates

### 2.1. Properties of Alginate

**Figure 2:**
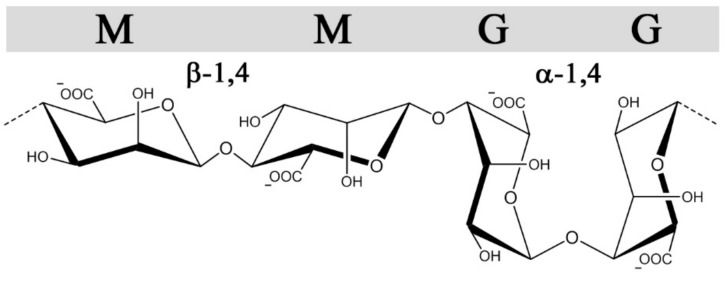
The structures and glycosidic bond conformations of β-d-mannuronic acid and α-l-guluronic acid in alginates.

Alginates, in contrast to GAGs, are produced in brown algae *(Pheaophyceae*), where they serve as structural polysaccharides, or in certain genera of gram-negative bacteria (*Azotobacter* and *Pseudomonas* sp.) where alginates as exocellular polysaccharides confer different protective functions and virulence factors [31]. Still, they share some structural features with heparin and heparin sulfate besides all being linear uronans. Alginates are copolymers of 1➜4-linked β-d-mannuronic acid (M) and α-l-guluronic acid (G) (Figure 2), which are C-5 epimers of each other, analogous to GlcA and IdoA in heparin/HS. Moreover, they are arranged in G-and M-blocks of various length interspaced with regions of alternating sequences (MG-block) akin to the sulfate-, IdoA-rich and undersulfated GlcA-rich regions(NS and NA domains, respectively) found in heparan sulfate [32]. Alginates form hydrogels through ionic cross-linking with divalent cations such as calcium, where the gel properties are largely influenced by the content and length of the G-blocks [33]. Alginate is synthetized as homopolymeric mannuronan, which in a post-polymerization step is converted into alginate by C-5 epimerization, similar to the GlcA→IdoA conversion in the biosynthesis of heparin and heparan sulfate. Due to the action of these post-polymerization epimerases, both GAGs and alginate possess non-random block sequential structures. Whereas the introduction of IdoA in heparin/HS provides conformational flexibility significant for protein interactions, the main effect of epimerization in alginates is the introduction of calcium binding G-blocks responsible for gel formation. The alginate-producing bacterium *Azotobacter vinelandii* expresses seven exocellular epimerases (AlgE1-7), which have been cloned and can be used to engineer alginates with compositionally homogeneous structures not found in nature [34].

Of note, the AlgE4 enzyme can be used to introduce an alternating sequence (poly-MG), similar to the basic backbone structure of heparin [35]. Enzymatic and chemical modifications of alginates have been extensively described in previous reviews, illustrating a great structural and functional versatility [36,37]. Alginate has been evaluated for numerous biomedical applications, primarily due to their gentle gelling conditions, including immunoisolation of cell transplants [38], slow-release systems [39], in vitro tissue engineering [40], and 3D-bioprinting [41]. Purified alginates are relatively inert toward cells and biomolecules, providing good biocompatibility but simultaneously discouraging favourable interactions with cell receptors and vital soluble factors that are characteristic of glycosaminoglycans in the extracellular matrix. Thus, great efforts have been made to functionalize alginates to provide a biomimetic environment, while maintaining their biocompatibility and gelling properties. One such strategy is by chemical sulfation, to emulate the structure of sulfated GAGs.

### 2.2. Synthesis and Characterization of Sulfated Alginates

**Figure 3:**
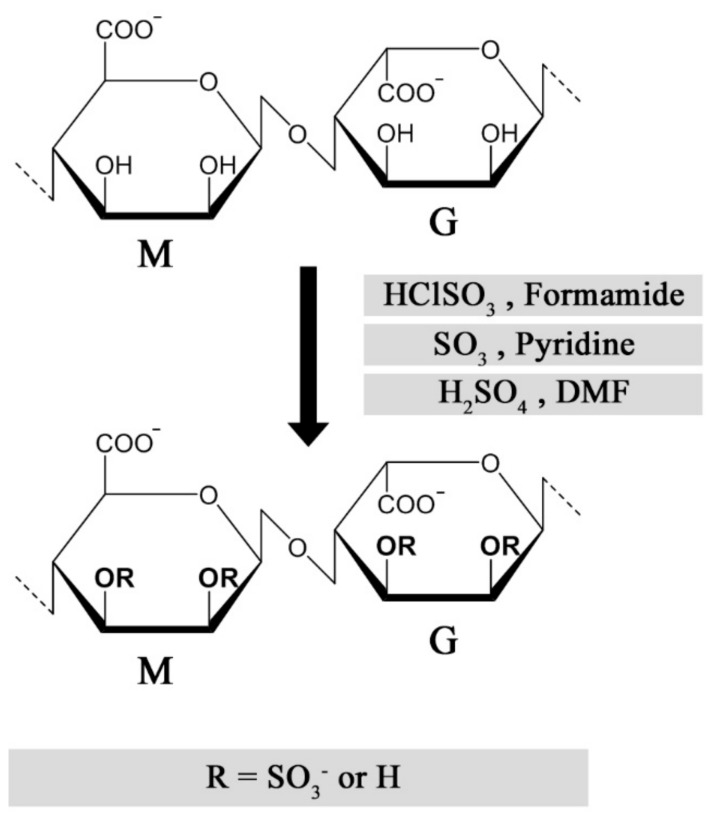
Published methods for chemical sulfation of alginate using different reagents [9,42,46,48].

Multiple strategies have been described for chemical sulfation of alginates (Figure 3). Huang and co-workers first employed chlorosulfonic acid (HClSO_3_) in formamide, resulting in a reported degree of sulfation (DS) of approximately 1.2 sulfate groups per monosaccharide. As a means to reduce the adverse effects from over-sulfation, the sulfated alginates were conjugated with quaternary amine groups, allowing a controlled reduction of anti-coagulating properties [42]. We found that the sulfation degree could be reproducibly tuned by varying the chlorosulfonic acid concentration, but the DS was found to reach a plateau around DS = 1.0–1.2, depending on the monosaccharide sequence and their relative solubility in acid [43,44]. Sulfation of dextran has been performed using sulfur trioxide (SO_3_) in pyridine, which was reported to result in a more homogeneous substitution compared with the HClSO_3_/formamide method [45]. This approach was used for alginate by Mhanna and coworkers, using a tetrabutylammonium (TBA) salt of alginate to increase solubility in pyridine [46], but was found to have challenges related to reproducibility of the sulfation degree in following studies. An alternative strategy employs a carbodiimide-H_2_SO_4_ intermediate reacting directly with alginate [47], or via the TBA salt of alginate in DMF [48]. One challenge with the described methods is the strong acidic conditions used to obtain a high sulfation degree, resulting in partial depolymerization of high-molecular weight alginates [47], whereas the relatively low solubility of alginate in acid can reduce reaction reproducibility and throughput. Fan and co-workers reported a novel strategy for the sulfation of polysaccharides under non-acidic conditions, obtaining a DS of approximately 2 sulfates/monosaccharide at optimal conditions [49]. Although the authors of this review were not able to sulfate alginates using the method as described, the procedure could have a large potential for preventing depolymerization and allowing a DS approaching that of heparin, if successfully established.

The molecular structure of sulfated alginates has been characterized primarily by utilizing Fourier-transform infrared (FTIR) spectroscopy, Nuclear magnetic resonance (NMR), and Mass spectrometry (MS)-based methods. In the FTIR spectrum, sulfation of the hydroxyl groups in alginate results in the appearance of a distinct peak corresponding to the symmetric stretching of the S=O bond [42,48]. However, this method provides little qualitative data and cannot clearly distinguish substitution at C-2 from C-3, nor the M from G in alginate. NMR can provide detailed structural data, but generates highly complicated spectra due to the heterogeneity in the substitution patterns and monosaccharide sequences of sulfated alginates. We generated alginates with homogeneous sequences (poly-M, poly-G, and poly-MG) with three specific degrees of sulfation using HClSO_3_, and employed various 2D NMR techniques to assign the 1D ^13^C spectrum and identify the substitution pattern for the distinct alginate sequences [43,44]. Consistent with previous studies, the sulfation followed a random substitution pattern, indicated by the increase in spectrum heterogeneity at low sulfation degrees [43]. Based on the NMR data, no apparent selectivity was found for the substitution of M/G, or C-2/C-3. This was also found by Zhao and co-workers in an early study on low-molecular weight sulfated guluronate, by NMR characterization [50]. Following this presumption, the sulfate groups are evenly distributed along the polysaccharide chains and not organized in domains of varying density (e.g., following alginate block sequences), in contrast with heparan sulfate [51]. The degree of sulfation (DS) can thus be expressed as the average number of sulfates per monosaccharide, and determined by biochemical methods or mass spectrometry-based elemental analysis [44,52].

Chemical sulfation of alginates results in a less heterogeneous substitution pattern compared with natural heparin and heparan sulfate, as the sulfate groups are presumably equally distributed between C-2 and C-3 of the mannuronic acid and guluronic acid moieties. This provides as mentioned a relatively homogeneous charge distribution along the polysaccharide chain, which can contribute to the study of structure-function relationships while reducing batch-to-batch variability. The published methods demonstrate limitations in terms of sulfation degree, as the substitution is restricted to the free hydroxyl group, while di-sulfated monosaccharides are presumably discouraged from steric effects. The different substitution pattern and lower charge density of sulfated alginates can in turn lead to more transient protein interactions, or the utilization of alternative interaction sites compared with heparin/HS. Additional strategies for C-6 sulfation can therefore be explored to emulate highly sulfated moieties [53]. Furthermore, monosaccharide-specific sulfation of alginate would carry a substantial benefit, as sulfation of primarily mannuronic acid would allow unimpaired cross-linking of guluronic acid blocks and a vast improvement in sulfated alginate gel strength and stability. This is, however, yet to be demonstrated, as there are great challenges in discerning between the monosaccharides chemically.

### 2.3. Chemical and Physical Properties of Sulfated Alginates

Introduction of a charged and relatively bulky substituent notably alters the chemical structure of alginates, which is of great relevance to their inherent properties in solution and in ionically-crosslinked hydrogels. Steric hindrance reduces rotation around the glycosidic bond, conferring a more extended and rigid conformation in polysaccharides [54]. The sulfate groups can further promote intramolecular charge repulsion, although this effect is reduced by the presence of sodium counter ions similarly to the carboxyl groups of alginate and is presumably negligible compared with steric effects. The precise effect of sulfation on alginate conformation in solution remains to be elucidated, and can be approached utilizing homogeneous sequences of alginates that have previously been studied in terms of chain extension and bond rotation [55]. The stiffness of the polysaccharide chain can influence interaction strengths with proteins, and thus the biological properties of the polysaccharides.

Sulfation generally has a deteriorating effect on the gelling ability of alginates, where the resulting gels have a lower stiffness and increased rate of swelling and destabilization compared with unmodified alginate [46,56]. Negatively charged sulfate groups associate with divalent cations, but disrupt the long G-blocks that are responsible for the cooperative binding of ions and forming of cross-linking junction zones in the gel network, as described in the “egg-box” model [57]. In a recent study, we characterized gels made exclusively from sulfated alginates with varying sulfation degrees, as well as the effect of combining highly sulfated alginate (DS = 1) in unmodified alginate gels at various proportions. Sulfated alginate alone (150 kDa) was found to form stabile gels with calcium up to a sulfation degree of approximately 0.4, whereas increasing the sulfation level required either the inclusion of unmodified alginate or utilization of gelling ions with a higher affinity for alginate (e.g., barium, strontium) [58]. An alternative strategy that has not yet been explored to the authors’ knowledge is covalent cross-linking between sulfated alginates, or to unmodified alginates, which can be a feasible approach where a higher gel stiffness is required. As highly sulfated alginates presumably only form transient cross-links within the hydrogel matrix, they diffuse out in the surrounding medium upon swelling of the gels at a higher rate than unmodified alginate, particularly at low molecular weight. Interestingly, a low amount (20%) of S-Alg mixed with unmodified alginate consistently demonstrated decreased stiffness but a lower swelling potential in saline than the alginate control, potentially due to a higher charge density, retention of gelling ions slowing the exchange with Na^+^, and osmotic influx of water [58].

The use of heparin analogues in hydrogels are of great interest for encapsulation of cells and proteins (e.g., in tissue engineering). Sulfated alginates demonstrate great potential with its inherent gelling capability and has, similarly to native alginate, a great versatility in properties and gelling conditions to allow the tuning of hydrogel characteristics toward specific applications. These include enzymatic engineering to increase gel strength [34], and the utilization of alternative cross-linking ions [59] and gelling techniques, for example, CaCl_2_ for immediate gelling versus CaCO_3_ and glucono-δ-lactone GDL for a gradual release of calcium in injectable solutions and in situ gelation [60].

### 2.4. Effects of Sulfated Alginates on the Coagulation Cascade

As heparin is most widely known and used due to its potent anticoagulating properties, it is of great interest to investigate whether similar effects can be achieved utilizing the structurally analogous sulfated alginates. Huang and co-workers studied the activated partial thrombosis time (APTT), thrombin time (TT), and prothrombin time (PT) in plasma, using sulfated algal alginates with an approximate DS of 1.0 sulfate per monosaccharide. Although heparin was not included as a control in the present study, sulfated alginates prolonged the APTT with increasing treatment concentrations while showing no significant effects on the TT and PT [42]. Conversely, Ma and co-workers showed a pronounced elevation of the TT increasing with the sulfation degree and concentration of sulfated alginate, whereas no comparison with heparin was made. The authors further demonstrated a procedure for coating a polyethersulfone membrane with sulfated alginate, resulting in prolonged coagulation compared with the non-coated membrane [47]. By hydrolysis and separation, Li and co-workers prepared low-molecular weight (6–7 kDa) alginates enriched in mannuronic acid or guluronic acid, and studied the anticoagulating effects of their sulfated derivatives. Similarly to the study by Huang, SA was found to increase the APTT compared to the saline control and the non-sulfated alginates, whereas a two- and eight-fold greater effect was observed for LMWH and heparin, respectively, at similar concentrations [61]. The results indicate that sulfated alginates may inhibit the extrinsic coagulation pathway through binding and sequestration and/or prevention of protease activity of upstream factors, but are unable to bind antithrombin selectively to inhibit tissue factor (TF)-mediated activation of Factor X, and thrombin. Lacking AT activation was also evident from the TT test where sulfated alginate was unable to prolong coagulation time in the presence of excess thrombin. Heparin does not deactivate TF directly in vivo, but induces secretion of an inhibitor (TFPI) from endothelial cells, resulting in a less pronounced effect on the PT in vitro [62]. To the authors’ knowledge, the release of TF has not been demonstrated for sulfated alginates or similar heparin analogues in in vivo models.

**Figure 4:**
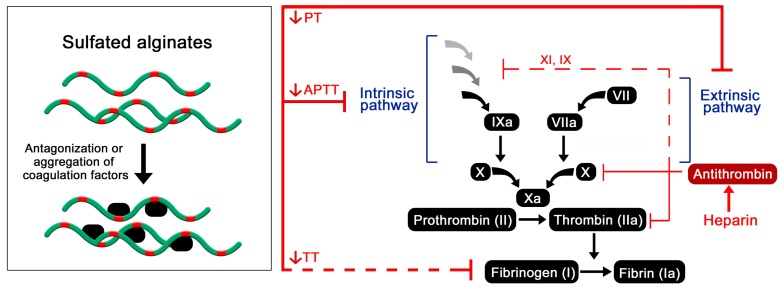
Postulated effect of sulfated alginates on the coagulation cascade resulting in prolonged coagulation time [42,47,49,50,61].

The mechanism behind the observed anticoagulating effects of sulfated alginates are still not clear, whereas the presented research does not strongly support the specific antithrombin activation that is characteristic of heparin. Presumably, the sulfated alginates non-specifically bind multiple coagulation factors, having a partial antagonizing or deactivating effect on the proteases or indirectly through other regulatory proteins (Figure 4). Alternatively, interaction outside the active sites may lead to aggregation and sequestration of precursors such as fibrinogen, as proposed for other heparin mimetics [63]. As the sulfated alginates were postulated to have a greater influence on the intrinsic coagulation pathway [42], interaction studies with individual coagulation factors can help further elucidate their effect on the coagulation cascade, by indicating whether the interactions are largely non-specific or if there is a selectivity for certain factors. Furthermore, platelet aggregation and activation are vital steps within the coagulation cascade, and should be studied for hydrogels or surface coatings of sulfated alginates to evaluate if they exhibit effects similar to heparin and other well-characterized heparin analogues [5,64].

### 2.5. Immunological Effects of Sulfated Alginates

The mechanisms behind the anti-inflammatory properties of heparin and similar sulfated polysaccharides are still not fully understood. Several studies demonstrate interactions with cytokines, chemokines, growth factors, and signalling cascade factors, potentially resulting in altered protein half-life, sequestration of ligands from their receptors, and/or prevention of proteolytic cleavage or conformational changes in proteins. The cellular response to inflammation can also be affected more directly by heparin, through association with adhesion proteins that mediate extravasation of leukocytes across the endothelium [29].

**Figure 5:**
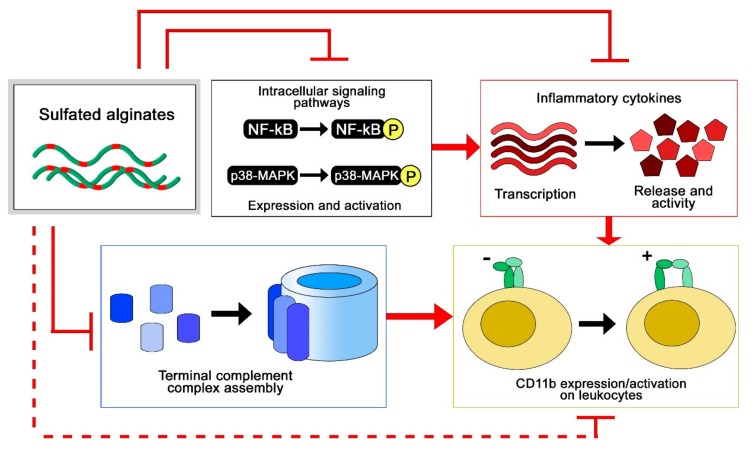
Anti-inflammatory effects of sulfated alginates from whole blood and cell culture models [58,65].

To study the anti-inflammatory properties of sulfated alginates, we employed two different model systems, where multiple anti-inflammatory effects were observed (Figure 5). In the first study, sulfated alginates were incorporated in alginate microspheres either as a secondary coat on polycation-coated microcapsules or mixed with non-sulfated alginate in uncoated microspheres, followed by incubation in whole human blood anti-coagulated with lepirudin [65]. Microspheres with sulfated alginates were found to attenuate the inflammatory response, by lowering the expression of several inflammatory cytokines, including interleukin (IL)-1β, TNF, and IL-8. The sulfated alginate gels were found to inhibit the complement cascade in blood, as well as in plasma in soluble form [44], indicating direct interaction with complement factors as previously demonstrated for heparin [19,66]. Sequestration of complement factors prevents assembly of the convertases and terminal complement complex, whereas we additionally demonstrated interaction between sulfated alginates and complement inhibitory Factor H, which can contribute to suppression of the complement cascade on microsphere surfaces [65]. Lastly, there is significant cross talk between the coagulation and complement cascades [20,67], where the previously described anticoagulating activities of sulfated alginates can have an indirect influence on the inflammatory response. Sulfated alginates were further found to reduce integrin alpha M (ITGAM/CD11b) expression on leukocytes, which can be attributed to indirect effects from sequestration of cytokines and complement factors. In the second study, human chondrocytes were encapsulated in alginate or sulfated alginate gels, prior to inflammatory induction with IL-1β. Chondrocytes in sulfated alginate gels demonstrated lowered expression of inflammatory and catabolic markers, as well as reduced nuclear factor-kappa b NF-κB and p38-mitogen activated protein kinase (MAPK)-mediated signaling compared with the alginate controls. Sulfated alginate was found to bind IL-1β, presumably sequestering the cytokines in the gel matrix and preventing induction of the encapsulated cells [58]. Similarly, Freeman and co-workers demonstrated binding to IL-6, further indicating that sulfated alginates can regulate cytokine activity as well as their expression [48]. Inhibition of cytokine activity through binding can appear to contradict with the potentiating effect of binding growth factors, and will depend on whether the interaction sites between sulfated alginates and proteins interfere with receptor binding, or if there is a co-receptor functionality as previously established for heparan sulfate and FGF [68]. Heparin has previously been demonstrated to inhibit leukocyte activity through associating with L- and P-selectins [22], which has not yet been demonstrated for sulfated alginates, but can potentially reveal additional anti-inflammatory effects in alternative model systems. As a model for chronic inflammation, Zhao and co-workers studied granuloma formation in rats, and found that ingested sulfated guluronate reduced the size of the granuloma [50]. The mechanisms behind this effect are still unclear, and sulfated alginates may act on multiple levels such as inflammation and coagulation pathways, and adhesion molecules [69,70].

### 2.6. Sulfated Alginates in Tissue Engineering and Drug Delivery

The use of heparin and other GAGs in biomaterials intended for long-term implantation is limited partly due to their rapid turnover in vivo. The exploration of more stable analogues has therefore been encouraged for a range of applications in tissue engineering and encapsulation of therapeutics. Cohen and co-workers initially reported sulfated alginates to associate with multiple heparin-binding growth factors, highlighting their potential to serve as a reservoir and a slow-release system for growth factors toward tissue cultivation and drug delivery [48]. Sulfated alginate hydrogels loaded with growth factors were further found to promote angiogenesis and blood perfusion in animal tissues, and have been formulated as injectable solutions for gelation in situ [71,72]. In a separate study, heterogeneous hydrogels with layered organization of specific growth factors were found to support compartmentalized differentiation of mesenchymal stem cells into osteoblasts and chondrocytes [73]. In a recent study, Ruvinov and colleagues proposed a model where the multiple heparin-binding proteins and sulfated alginate chains spontaneously assemble into nanoparticles with a fiber-like structure and a net negative surface charge, which can subsequently be immobilized in injectable hydrogels such as unmodified alginates or various nanoparticle formulations [74]. This demonstrates a great potential and versatility for formulating hydrogel- and nanoparticle-based delivery of heparin-binding proteins, whereas modifications to the structure and sequence of the sulfated alginates can additionally contribute to the release rate by tuning the interaction strength with various proteins [43,44]. In addition to providing prolonged delivery of growth factors through their associative retention, the binding of sulfated alginate has been demonstrated to protect the proteins from proteolytic cleavage by trypsin [74], which is one of the postulated effects of heparin and other sulfated glycosaminoglycans in regulating cell signaling.

**Figure 6:**
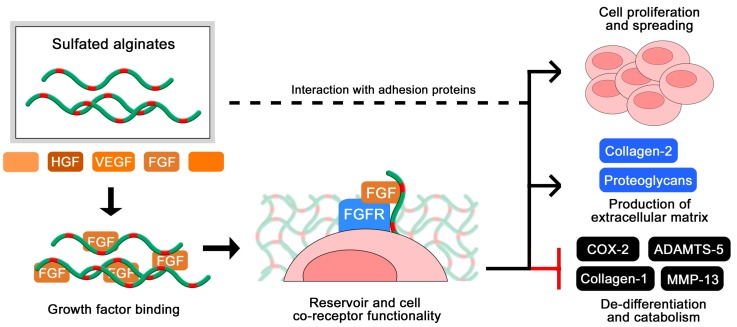
Sulfated alginates bind multiple growth factors [48], and have an inductive effect in chondrocyte cultivation [46,56,58].

Zenobi-Wong and colleagues initially employed sulfated alginate hydrogels for the cultivation of cartilage, and found that the gels promoted proliferation and prolonged viability of the chondrocytes (Figure 6). Furthermore, the sulfated alginates were able to sustain the cartilaginous phenotype over long culture times, demonstrated by a high degree of collagen 2 expression and repressed collagen 1 expression [46]. The inductive effect on chondrocyte proliferation was attenuated by blocking beta1 integrins, indicating that the sulfated alginates interact (presumably indirectly) with integrins and potentially other adhesion proteins on the cell surface to support anchorage and migration. The chondrogenic effects were additionally related to a high degree of FGF retention in the hydrogels, where the sulfated alginates are proposed to act as a co-receptor to the cellular FGF receptor, analogous to heparan sulfate in vivo [56]. This was indicated by the ability of sulfated alginates to restore FGF-mediated proliferation of HS-deficient BaF3 cells [56], and was later reproduced by Li and co-workers utilizing additional growth factors and different sequences of sulfated alginate [61]. Due to their beneficial effects on chondrocyte proliferation and phenotype maintenance, sulfated alginates have further been evaluated as a component in bioinks for 3D-bioprinting, including nanocellulose fibers to retain the shape of printed structures prior to ionic crosslinking [75].

Whereas the degradation of sulfated alginates has not yet been studied in vivo, alginates are not depolymerized by any known enzyme in mammals. Sulfated alginates may thus provide more stable hydrogels for long-term implants, where alteration of the sulfation degree, monosaccharide sequence, and gelling conditions can tune properties such as swelling and porosity, degradability, and retention of matrix-binding proteins. Despite a low inflammatory response from the innate immune system, in vivo experiments in certain rat models and primates have uncovered a fibrotic reaction to pure alginate implants, which will presumably occur in human subjects as well and impair the function of the hydrogels and encapsulated cells. As elaborated in the previous section, sulfated alginates show potent anti-inflammatory properties, which can reduce immunological rejection and fibrosis toward long-term implants and potentially overcome these challenges with alginate-based biomaterials. The encapsulation of cells in biomimetic matrices is a highly relevant approach for generating more advanced in vitro tissue models, and for clinical tissue engineering including matrix-assisted cell implantation and injectable matrices such as supporting scaffolds for tissue repair [76,77].

### 2.7. Structure-Function Relationships in Sulfated Alginates

The conformations of M and G are ^4^C_1_ and ^1^C_4_, respectively, with glycosidic bonds that are di-equatorial, di-axial, or equatorial-axial for MM, GG, and MG, resulting in varying orientations of the sulfated C2 and C3 hydroxyl groups (Figure 2). The sequences vary in their extension and rigidity, where the GG sequences have a more compact conformation with higher charge density per unit length compared with MM. The MG sequence was initially demonstrated by Smidsrød and colleagues to have a more flexible backbone compared with MM and GG [55], which is also supported by its higher solubility in water at low pH [78]. Similar to heparin, structural and conformational properties, alongside the negative charge density, can influence the interaction strength between sulfated alginates and proteins, emphasizing the importance of characterizing structure-function relationships.

Oligosaccharides of sulfated alginates have been utilized to study the minimal degree of polymerization (DP) required for binding to proteins. In one of our studies using a sulfated alternating alginate sequence (S-MG, DS~0.9), a minimum length of 8-mer was required for significant interaction with HGF, whereas 14-mers approached the interaction strength of poly-S-MG (DP = 80) and LMWH. For FGF-2, a low degree of interaction was observed for 6-mers of S-MG, whereas the 14-mers did not show the efficacy of poly-SMG. Overall, the S-MG samples showed a lower degree of interaction with FGF-2 compared with HGF, and did not approach the efficacy of heparin. Liu and co-workers prepared and sulfated (DS~1.5) M-rich oligosaccharides obtained from hydrolysis and separation of algal alginate, and studied the binding interaction to HIV envelope protein gp120, as a potential anti-HIV therapy. It was demonstrated that a minimal length of an 8-mer was required for interaction, whereas multivalent interactions were observed for >15–16 mers as well as a higher binding affinity compared with heparin [79]. As alginates can be reproducibly hydrolysed and separated into low-disperse fractions based on size and monosaccharide composition, optimizing the molecular weight of samples can potentially contribute to reducing adverse effects in therapeutic applications, similarly to the use of UFH/LMWH/ULMWH.

To assess the impact of the alginate monosaccharide sequence, we initially utilized homogeneous sequences (poly-M, poly-G, and poly-MG) of sulfated alginates with similar sulfation degrees and analysed the interaction with HGF [44]. Here, it was found that at high sulfation degrees (DS~1) the efficacy of the varying sequences was similar, whereas at intermediate sulfation degrees (DS~0.5) the interaction strength increased in the order of poly-M < poly-G < poly-MG. This points towards an influence of chain flexibility as the highly sulfated alginates are presumed to display a more rigid conformation, thus reducing the effect of backbone flexibility in the unmodified alginate sequences. To further investigate the effect of chain flexibility, we performed a periodate oxidation of sulfated poly-M alginates, causing hexuronic ring opening in non-sulfated monosaccharides and an introduction of flexible junction zones [43]. This resulted in increased interaction strength with HGF and FGF-2, correlated with the oxidation degree of the sulfated alginates. The importance of chain flexibility is expected to vary between proteins of different sizes and surface patterns of basic amino acid residues. It was discovered that sulfated poly-M alginates interacted more strongly to FGF-2 than the sulfated poly-MG alginates, while periodate oxidation had a smaller impact on interaction strength compared with that for HGF, indicating that the monosaccharide structure and charge orientation contributes to interaction strength alongside chain flexibility. This was again demonstrated by Li and co-workers, who found that sulfated G-rich oligosaccharides were more effective that M-rich oligosaccharides at prolonging coagulation time, and at mediating FGF-8 signalling in BaF3 cell cultures [61]. As over-sulfated alginates may exert a high degree of non-specific and potential adverse effects, chemo-enzymatic engineering of the backbone may provide a versatile tool to improve interaction strengths and potentially confer increased ligand selectivity compared to sulfation alone, similarly to the role of IdoA in heparin and heparan sulfate.

## 3. Conclusions and Future Directions

From the present status of knowledge, sulfated alginates show in particular promise for cell immobilization and tissue engineering applications, as presented through the works of Zenobi-Wong and Cohen with their respective groups. The sulfated alginates act as analogues of cell surface GAGs in mediating growth factor signalling, while creating a biomimetic physical environment for the proliferation and migration of cells, extracellular matrix deposition, and tissue maturation. Similarly, the sulfated alginate matrix may provide a reservoir for growth factors, where the affinity toward specific growth factors may be altered through chemo-enzymatic engineering of the alginate, for a tuned and sustained delivery to tissues. There have further been demonstrated anti-inflammatory properties in sulfated alginates, which can aid in matrix-assisted cell- and tissue transplantations by suppressing pro-catabolic and inflammatory responses from surrounding tissues and in encapsulated cells. However, additional in vivo studies to assess gel stability, immunological and fibrotic responses, and implant survival are required. Non-coagulating heparins have previously been evaluated for anti-inflammatory and anti-cancer therapeutics, highlighting an additional potential application for heparin analogues with customizable pharmacokinetic and -dynamic properties in treating acute and chronic inflammatory conditions.

Several studies on sulfated alginates have focused on their anti-coagulant properties. However, there are no clear indications of specific antithrombin activation, where substantially higher concentrations of sulfated alginates are required to approach the efficacy of heparin. While sequences of sulfated alginate with a higher affinity for antithrombin can potentially be generated through chemo-enzymatic engineering, emulation of the specific heparin pentasaccharide may prove challenging. As an intravenously administered drug with rapid onset, heparin benefits from a short half-life. Sulfated alginates are presumably eliminated through renal excretion alone, which is a slower mechanism compared to depolymerization, where extended activity may lead to a severe drop in blood pressure and additional adverse effects. The use of sulfated alginates as anticoagulants can therefore show greater promise as a coating for medical devices and biomaterials, where a high surface stability is desirable and the additional anti-inflammatory effects can potentially reduce fibrosis or immunological rejection of implanted devices or materials.

Chemical sulfation of alginates allows for the simple and reproducible synthesis of heparin-like molecules in large batches and at low cost. However, much work remains to explore novel strategies for milder and more selective syntheses, to further characterize and understand their interaction with heparin-binding proteins, and to evaluate the potential of sulfated alginates toward novel biomedical applications. Compared to GAGs and other heparin analogues, the utility of sulfated alginates is tied to their gelling capability and their structural customizability, allowing tuning of physical and biological properties, and bioavailability. The majority of studies have employed commercial algal alginates and have performed sulfation of C2 and C3, whereas exploring non-conventional sequence patterns (alternative sources, enzymatic engineering), novel sulfation strategies (M/G preference, C6-sulfation), and conjugation to other functional groups can provide new properties and potentially improved selectivity of protein interactions. Ideally, a sulfated alginate oligosaccharide library with defined modification patterns can be established and applied to gain a deeper understanding of their structure-function relationships. In conclusion, sulfated alginates have unique characteristics as heparin analogues, and while they are relatively unstudied, they demonstrate a wide range of biological properties and a structural versatility that can provide novel biomedical applications and a deeper understanding of the biological functions of sulfated glycosaminoglycans.
